# Late-Onset Metastatic Malignant Spindle Cell Tumour Presenting with Massive Intra-Abdominal Haemorrhage

**DOI:** 10.1155/2020/8812647

**Published:** 2020-12-16

**Authors:** Pamathy Gnanaselvam, Malintha Lahiru, Mariathas Priatharshan, Umesh Jayarajah, Kathirvetpillai Kopinath

**Affiliations:** ^1^Department of Surgery, National Hospital of Sri Lanka, Colombo, Sri Lanka; ^2^Faculty of Medicine, University of Colombo, Colombo, Sri Lanka; ^3^Department of Pathology, National Hospital of Sri Lanka, Colombo, Sri Lanka

## Abstract

Primary spindle cell sarcoma is a rare tumour. The presentation of acute intra-abdominal bleeding from a metastatic spindle cell tumour has not been previously reported. We report a case of a 40-year-old woman with a history of curative resection of the medial compartment of the right thigh for spindle cell sarcoma presenting with an acute onset abdominal pain and haemorrhagic shock after 5 uneventful years. Emergency exploratory laparotomy was conducted that revealed a retropancreatic mass which had ruptured along its inferior border. Histological evaluation revealed a metastatic deposit of the spindle cell sarcoma. In cases of spontaneous abdominal haemorrhage, it is important to consider the possibility of a ruptured metastatic deposit among the differentials especially in patients with a history of malignancies. Moreover, this is the first reported case of metastatic malignant spindle cell sarcoma presenting with intra-abdominal haemorrhage.

## 1. Introduction

Primary spindle cell sarcoma is a rare soft-tissue tumour that lacks a specific cell line of differentiation and is among the least reported tumours in the world [1]. Being primarily a soft-tissue tumour, it commonly arises in the layers of connective tissue such as under the skin, in between muscles, and surrounding organs. Due to the scarcity of reported literature, it poses a diagnostic and therapeutic challenge [2]. Even though the exact aetiology is poorly known, genetic predisposition and underlying inflammatory process are possible risk factors of this disease [3]. Although the majority of these tumours are found in the long bones of the body, such as the femur and bones of the knee joint, tibia, and humerus, spindle cell sarcoma can arise in any area of the body [4, 5]. As such, the range of potential differential diagnoses even in rare locations includes osteosarcoma, rhabdomyosarcoma, leiomyosarcoma, squamous cell carcinoma, and solitary fibrous tumours [6–10]. Following successful treatment of their primary site, distant metastases have been seen in 25% of the patients [11]. Metastatic spindle cell sarcoma presenting with massive intra-abdominal haemorrhage is extremely rare, and such cases have not been reported in the literature. In this case report, we describe a 40-year-old female with a history of curative medial compartment resection of the thigh for a malignant spindle cell tumour later presenting with massive intra-abdominal haemorrhage due to metastatic deposits.

## 2. Case Presentation

A 40-year-old previously healthy woman underwent a curative resection of the medical compartment of the right thigh for a malignant spindle cell tumour. Due to clear resection margins and absence of any metastatic deposits in imaging, she was not given any adjuvant therapy. She was followed up for 5 years, and she was otherwise well and asymptomatic without any evidence of local recurrence.

After a period of 5 years, she developed an acute onset generalized severe abdominal pain and collapsed. She did not have any external bleeding manifestations such as haematemesis or melaena. There was no history of abdominal trauma. She was immediately admitted to the emergency department, and she was found to be in class III haemorrhagic shock with a pulse rate of 125 beats per minute and a blood pressure of 90/60 mmHg. Her abdomen was distended and diffusely tender. She was immediately resuscitated with intravenous crystalloid boluses and blood transfusion. Her serum beta-Human Chorionic Gonadotropin (beta-HCG) was negative, excluding a ruptured ectopic pregnancy. Her full blood count revealed a haemoglobin level of 5 g/dl, and a massive transfusion protocol was initiated. Her other basic investigations and clotting profile were within normal limits. Despite resuscitation, her vital parameters did not improve and her abdominal girth was gradually increasing. The abdominal ultrasound scan revealed moderate amount of free fluid with a mass around the proximal part of the pancreas. There was no evidence of abdominal aortic aneurysm, and other solid organs were normal. Hence, a decision was taken to proceed with an emergency exploratory laparotomy. A computed tomography was not done as the patient was haemodynamically unstable.

A midline laparotomy was performed. There was 4.5 litres of haemoperitoneum, and a linear tear in the gastrocolic omentum with a haematoma in the lesser sac. Active bleeding was noted from a nidus which was situated posterior to the body of the pancreas, and it had ruptured through the inferior border of the pancreas. The rest of the pancreas and other abdominal organs looked normal. Bleeding was controlled by ligation and local application of haemostatic agents. A biopsy was taken for histopathological analysis. The pancreas and the rest of the organs were macroscopically normal.

**Figure 1:**
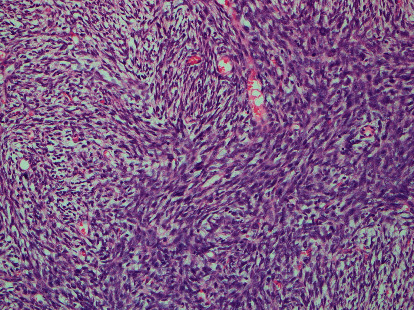
H&E 100x showing microscopically infiltrating tumour composed of fascicles of spindle cells with moderate to severe nuclear pleomorphism and increased mitotic activity in a prominent storiform pattern.

The histological analysis revealed an infiltrating tumour composed of fascicles of spindle cells with oval, vesicular nuclei with the scanty eosinophilic cytoplasm. There were areas containing cells with moderate to severe nuclear pleomorphism and increased mitotic activity (13/10 high power field). The prominent storiform pattern was evident (Figure 1). The focal haemangiopericytomatous vascular pattern was present. There was no tumour necrosis. Entrapped atrophic pancreatic acini were seen within the tumour. CD 99 and PS 3 markers showed moderate cytoplasmic positivity and nuclear positivity, respectively. SMA showed nonspecific positivity. CD 34, BCL 2, S100, EMA, CK7, CK19, and Desmin were negative. Therefore, the diagnosis was compatible with an undifferentiated spindle cell sarcoma involving the pancreas. Furthermore, the histological patterns were compatible with a metastatic deposit of the previously diagnosed spindle cell sarcoma.

**Figure 2:**
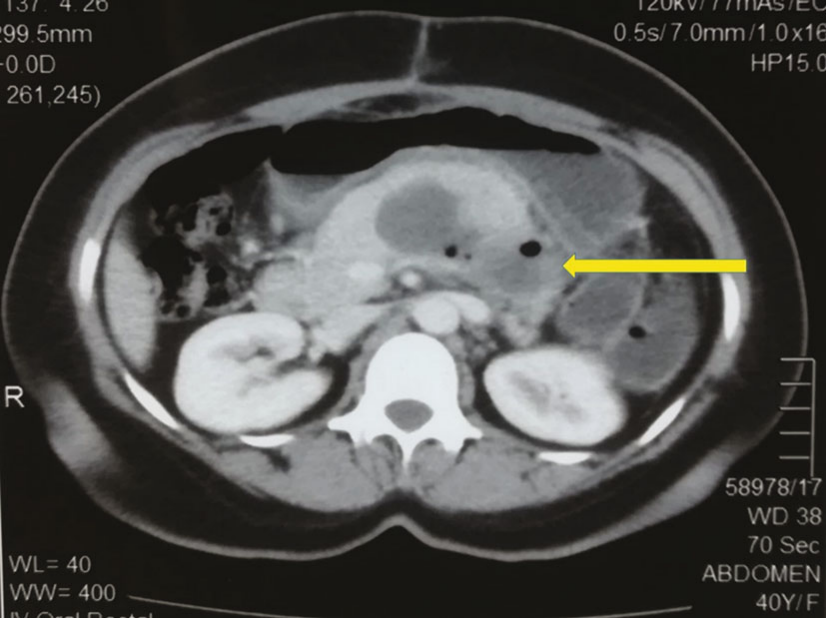
Postoperative computed tomography image showing a 7 × 4.8 × 4.9 cm mass lesion with a thin wall containing hypodense material, attached to the inferior border of the pancreas.

Following surgery, the patient was managed in the intensive care unit for 4 days, and thereafter, the patient recovered and was discharged on day 7. She was offered pancreatectomy with excision of the tumour but she refused surgery. She was referred to the oncologist and was commenced on adjuvant chemotherapy. She received a course of Ifosfamide and Doxorubicin. Contrast-enhanced computed tomography was performed before the commencement of chemotherapy which showed a 7 × 4.8 × 4.9 cm mass lesion with a thin wall containing hypodense material, attached to the inferior border of the pancreas (Figure 2). There were no other metastatic lesions identified. She was given 6 months of chemotherapy and finally succumbed to the disease 18 months after the presentation.

## 3. Discussion

Spindle cell neoplasms encompass a wide diversity in clinicopathologic and biological heterogeneity [12]. Primary spindle cell sarcoma is very rare; hence, it is the least reported [1]. Being a connective tissue tumour, it may arise from any tissue in the body [2]. As the literature is infrequent, it poses a considerable diagnostic and therapeutic challenge [13, 14].

In the reported literature, primary tumour has been treated aggressively with surgical therapy, along with adjuvant chemoradiotherapy in high-risk patients. In a population-based analysis including 3299 patients, around 60% of the overall cases were treated with surgery alone, and despite different surgical modalities, they had a relatively favourable disease-specific and overall survival rates compared to the nonsurgical group [2].

Similarly in our case, the patient underwent curative resection of the medial compartment alone as a treatment modality without conferring the patient to adjuvant therapy. Even though there was no evidence of metastatic disease at the time of surgery, the patient presented with complications of metastatic disease after 5 years.

Metastatic tumour presenting with massive intra-abdominal bleeding is a relatively rare phenomenon, and spindle cell sarcoma presenting with such phenomena is even rarer. Bhardwaj et al. describes a case of metastatic squamous carcinoma of the lung presenting with upper gastrointestinal bleeding [15]. Joy et al. describes a case of lobular breast carcinoma presenting with upper gastrointestinal bleeding [16]. Renal cell carcinoma has also been reported to present with upper gastrointestinal bleeding due to metastatic deposits [17]. To the authors' knowledge, the presented case is the first reported occurrence of massive intra-abdominal bleeding secondary to metastatic to spindle cell sarcoma.

In our patient, proper preoperative work-up was not possible as the presentation was an emergency, and the priority was to achieve haemostasis and stabilization rather than tumour excision. Due to the haemodynamic instability and the difficult anatomical location of the tumour, curative dissection was not attempted, and a biopsy was taken to confirm the diagnosis. Retroperitoneal metastasis is very rare in this tumour and mostly occurs in the lungs because of haematogenous spread.

Although rare, it is important to consider the possibility of a ruptured metastatic deposit among the differential diagnoses in an event of a spontaneous abdominal haemorrhage. Obtaining a detailed history is important to detect previous history of malignancies that could give rise to this rare complication. Furthermore, this may be the first presentation of a tumour in an otherwise healthy person.

## 4. Conclusion

We describe a middle-aged female with a history of curative medial compartment resection of the thigh for a malignant spindle cell tumour later presenting with massive intra-abdominal haemorrhage due to metastatic deposits. In cases of spontaneous abdominal haemorrhage, especially in patients with a history of malignancies, it is important to consider the rare differential diagnosis of a ruptured metastatic deposit.

## Data Availability

All underlying data supporting the results of the study are included in the manuscript.
